# Performance of prostate cancer recurrence nomograms by obesity status: a retrospective analysis of a radical prostatectomy cohort

**DOI:** 10.1186/s12885-018-4942-0

**Published:** 2018-11-03

**Authors:** Charnita Zeigler-Johnson, Aaron Hudson, Karen Glanz, Elaine Spangler, Knashawn H. Morales

**Affiliations:** 1 Thomas Jefferson University, 834 Chestnut Street, Suite 311, Philadelphia, PA 19107 USA; 2 University of Washington, Seattle, WA USA; 3 University of Pennsylvania, Philadelphia, PA USA

**Keywords:** Biochemical recurrence, CaPSURE/CPDR score, Kattan score, Obesity, Prostate Cancer

## Abstract

**Background:**

Obesity has been associated with aggressive prostate cancer and poor outcomes. It is important to understand how prognostic tools for that guide prostate cancer treatment may be impacted by obesity. The goal of this study was to evaluate the predicting abilities of two prostate cancer (PCa) nomograms by obesity status.

**Methods:**

We examined 1576 radical prostatectomy patients categorized into standard body mass index (BMI) groups. Patients were categorized into low, medium, and high risk groups for the Kattan and CaPSURE/CPDR scores, which are based on PSA value, Gleason score, tumor stage, and other patient data. Time to PCa recurrence was modeled as a function of obesity, risk group, and interactions.

**Results:**

As expected for the Kattan score, estimated hazard ratios (95% CI) indicated higher risk of recurrence for medium (HR = 2.99, 95% CI = 2.29, 3.88) and high (HR = 8.84, 95% CI = 5.91, 13.2) risk groups compared to low risk group. The associations were not statistically different across BMI groups. Results were consistent for the CaPSURE/CPDR score. However, the difference in risk of recurrence in the high risk versus low risk groups was larger for normal weight patients than the same estimate in the obese patients.

**Conclusions:**

We observed no statistically significant difference in the association between PCa recurrence and prediction scores across BMI groups. However, our study indicates that there may be a stronger association between high risk status and PCa recurrence among normal weight patients compared to obese patients. This suggests that high risk status based on PCa nomogram scores may be most predictive among normal weight patients. Additional research in this area is needed.

## Background

Prostate cancer (PCa) is a major public health burden with few confirmed risk factors [1]. Obesity, a common and potentially modifiable risk factor, affects approximately 20% to 30% of individuals with PCa [2–4] and appears to increase the risk of advanced PCa at diagnosis and risk for treatment failure [4–12]. Obese men also may be at increased risk for PCa-specific and/or overall mortality [9, 10], however not all studies support this relationship [3]. Therefore, it is important to understand the impact of obesity on tools used to aid in prognostication and guidance of treatment decisions.

Clinicians determine which cancer patients should receive definitive treatment by relying on nomograms. Nomograms are models that can predict PCa recurrence up to 10 years after the time of treatment with relatively high accuracy (71–79%) compared to other risk grouping methods [13, 14]. The Kattan nomogram, one of the most commonly used tools for predicting risk for PCa recurrence [15], includes information about year of radical prostatectomy, positive surgical margins, extracapsular extension, seminal vesicle invasion, lymph node invasion, primary and secondary Gleason grades, and preoperative PSA [16]. The CaPSURE/CPDR recurrence equation includes information about patient race, sigmoid transformed PSA, pathology stage (including organ confined vs. extracapsular disease), and post-operative Gleason sum [17].

Despite the widespread use of the Kattan and CaPSURE/CPDR as prognostic tools, it remains unclear how well they predict disease recurrence in obese patients of different PCa risk groups. We analyzed the predicting abilities of the 2 nomograms for PCa recurrence by obesity status. We hypothesized that the predicting ability of both nomograms would differ by body mass index (BMI) group depending on PCa risk status.

## Methods

This secondary data analysis was conducted using a sample of 2088 radical prostatectomy patients from the Study of Clinical Outcomes, Risk, and Ethnicity (SCORE) [18, 19]. Incident PCa cases were recruited at the University of Pennsylvania Health System (UPHS, Philadelphia, PA). Patients were excluded from this analysis based on the following criteria: not African American or Caucasian (*N* = 39), missing all variables used to calculate the risk score leaving no information for the imputation model (*N* = 13), missing information needed to calculate survival time (*N* = 213), missing information required to calculate BMI (*N* = 259), did not have surgery date recorded (*N* = 55), and status of cancer recurrence unknown (*N* = 217). After patients were excluded from the analysis, there was a final sample of 1576 patients. Informed consent was obtained from all individual participants included in the study under a protocol approved by the Institutional Review Board at the University of Pennsylvania and that conformed to provisions of the Declaration of Helsinki.

Obesity was defined using BMI categories. Patients with a BMI greater than 30 kg/m^2^ were classified as obese, patients with a BMI between 25 kg/m^2^ and 30 kg/m^2^ were classified as overweight. Eleven men met criteria for underweight (BMI < 19.5 kg/m2), but due to the small number, they were grouped with the normal weight category. Prostate cancer recurrence was defined as two PSA levels recorded above 0.2 ng/ml at any time after initial radical prostatectomy treatment. A total of 550 (35%) patients were missing information necessary for score calculation, so multiple imputation methods were used to prevent unnecessarily discarding data. Specifically, 476 were missing primary pathology Gleason grade, 511 were missing secondary pathology Gleason grade, and less than 40 were missing pathology Gleason score, PSA, extracapsular extension, surgical margin, seminal vesicle invasion, or lymph node involvement. Baseline and clinical characteristics were compared between the group with complete data and the group with missing data using T-test or Chi-square test as appropriate. A multiple imputation model was implemented within SAS v9.3 using a fully conditional specification (FCS) method for arbitrary missing data patterns [20]. The process, implemented in two phases, started with a fill-in phase where the values are filled-in one variable at a time and serve as starting values for the imputation phase. The imputation phase used the discriminant function method for binary covariates and regression method for continuous covariates. The multiple imputation process, which produced 5 imputed datasets after 20 burn-in samples per imputation, included all variables used to calculate the scores in addition to the clinical Gleason score. Trace plots were evaluated to ensure there were no systematic trends in the burn-in samples prior to the imputation sample.

Patients were categorized into recurrence risk groups using the Kattan nomogram and the CaPSURE/CPDR recurrence equation. Each risk score was considered in separate analyses. Using the Kattan nomogram, patients with a score between 50 and 100 were defined to be at a high risk of recurrence, patients with a score between 10 and 50 were defined to be at a medium risk of recurrence, and patients with a score less than 10 were defined to be at a low risk of recurrence. Using the CaPSURE/CPDR equation, patients with a score greater than 16.7 were defined to be at a high risk, patients with a score between 7.1 and 16.7 were defined to be at a medium risk, and patients with a score less than 7.1 were defined to be at a low risk [17].

Relationships between BMI groups and patient characteristics at time of diagnosis were assessed using Chi-square tests or Fisher’s exact test and ANOVA or Kruskal-Wallis test, depending on the distribution of the variables. Age, PSA and years since surgery were collected by medical record abstraction. Marital status, race, education, smoking and exercise data were collected by patient survey. All baseline characteristics were included because they have been associated with obesity and/or prostate cancer outcomes in previous studies. Bivariate relationships between time to PCa recurrence and patient characteristics were assessed using Cox Proportional Hazards models. Variables that were statistically significantly related to both obesity group and PCa recurrence at the 0.10 level were included in the final models.

Cox Proportional Hazards models were used to examine the association between time to recurrence and predicted risk recurrence group with potential effect modification by BMI group. A separate model that contained a main effect for obesity classification and the interaction between risk group and obesity group was developed for each risk score. Since the risk groups are arbitrary, models using the continuous risk scores were also fit. Descriptive analyses, multiple imputation, and Cox models were implemented in SAS v 9.4 (SAS Institute Inc., Cary, NC).

The diagnostic accuracy in comparison to a gold standard, in this case the observed PCa recurrence, was quantified for each continuous risk score by obesity category using a nonparametric estimimate of the area under the receiver operating characteristic curve (AUC) [21]. The equality of the AUC across risk scores were compared using a nonparametric test [22]. AUC analyses were implementd in Stata v 15 (StataCorp, College Station, TX).

## Results

In comparing men with complete data to men is at least one missing item, time since surgery was statistically different (*p* < 0.001) between those with complete data (mean = 10.5 months, standard deviation = 5.1) and those requiring imputation (mean = 15.3 months, sd = 5.5). African American men had lower rates of missing data compared to Caucasian men (21% vs 38%, Chi Square(df = 1) = 33.83, p < 0.001). Current, former, and never smokers also differed in rates of missing data (42.2%, 35.7%, and 29.1%, respectively; Chi Square(df = 2) = 11.83, *p* = 0.003). The analysis sample after imputation included a total of 1576 patients (383 obese, 820 overweight, and 373 normal weight).

Baseline characteristics and a summary of their relationship with PCa recurrence and obesity groups appear in Table 1. The follow-up time at which 25% of the men experienced prostate cancer recurrence was 47 months for obese patients, 70 months for overweight patients, and 121 months for normal weight men. In bivariate analyses, an increase risk of PCa recurrence was associated with increasing age and Arican American compared to Caucasian race. Obesity status was associated with age, years since surgery, PSA mass, exercise, smoking status, and race. The groups differed in pathology with a higher proportion of obese men having extracapsular extension, seminal vesicle invasion, lymph node involvement, higher pathological Gleason score, positive surgical margins, and adjuvant therapy compared to the same in normal weight men (Table 2). Age and race, associated with both obesity group and PCa recurrence, were included in adjusted models for the Kattan risk score. The models for CaPSURE/CPDR included age and factors from the Kattan risk score that are not included CaPSURE/CPDR score (Table 2: seminal vesicle invasion, lymph node involvement, adjuvant radiotherapy, and time since surgery).

**Table 1:** Baseline characteristics and the association with obesity status and time to PCa recurrence

Patient Characteristic	Obese (*n* = 383)	Overweight (*n* = 820)	Normal Weight (*n* = 373)	F or Chi-Square *p*-value	Hazard Ratio (95% CI)
Age in Years (Mean, SD)	58.6, 6.4	59.6, 6.8	60.0, 7.2	.014	1.009 (0.991,1.027)
Years Since Surgery (Mean, SD)	11.4, 5.8	12.2, 5.6	12.8, 5.9	.004	0.988 (0.965,1.011)
PSA mass μg (mean, SD)	89.8, 95.6	65.5, 65.8	50.0, 56.9	< 0.001	1.004 (1.004, 1.005)
Exercised, N (%)				.009	
More than once per week	144 (56.0)	344 (64.4)	157 (69.2)		1.084 (0.770,1.527)
Once per week or less	113 (44.0)	190 (35.6)	70 (30.8)		Ref
Marital status, N (%)				.161	
Married	269 (79.1)	623 (83.6)	276 (83.6)		0.964 (0.692,1.342)
Unmarried	71 (20.9)	122 (16.4)	54 (16.4)		Ref
Race, N (%)				< 0.001	
African American	103 (26.9)	160 (19.5)	59 (15.8)		1.564 (1.210,2.021)
Other	280 (73.1)	660 (80.5)	314 (84.2)		Ref
Smoking status, N (%)				.087	
Current	31 (8.8)	56 (7.4)	41 (12.0)		1.440 (0.926, 2.240)
Former	161 (45.9)	334 (43.9)	135 (39.6)		1.244 (0.962,1.608)
Never	159 (45.3)	371 (48.8)	165 (48.4)		Ref
Education, N (%)				.165	
College or higher	213 (61.9)	510 (66.1)	239 (68.7)		0.845 (0.657,1.086)
Less than college	131 (38.1)	262 (33.9)	109 (31.3)		Ref

Summary statistics are provided prior to multiple imputation. Column percentages exclude missing data: PSA mass (*n* = 185), exercise (558), marital status (161), smoking status (123), education (112)

**Table 2:** Clinical characteristics used in the calculation of the risk score and the association with obesity status

Clinical Characteristics	Factor in risk score calculation		Obese (*n* = 383)	Overweight (*n* = 820)	Normal Weight (*n* = 373)	F or Chi-Square *p*-Value
	Kattan	CaPSURE/CPDR				
Pre-surgery PSA ng/mL, mean, SD	X	X	6.3, 6.2	6.1, 5.7	6.1, 6.5	0.861
Pathological staging						
Extracapsular Extension, N (%)	X	X	125 (32.6)	204 (24.9)	87 (23.3)	0.005
Seminal Vesicle Invasion, N (%)	X		37 (9.7)	43 (5.2)	29 (7.8)	0.016
Lymph Node, N (%)	X		6 (1.6)	6 (0.7)	0 (0.0)	0.045
Pathological Gleason Score		X	6.7, 0.8	6.5, 0.8	6.5, 0.9	0.001
Pathological Gleason Grade1 ≥ 4, N (%)	X		57 (14.9)	87 (10.6)	34 (9.1)	0.045
Pathological Gleason Grade 2 ≥ 4, N (%)	X		145 (37.9)	252 (30.7)	101 (27.1)	0.019
Surgery Year, range	X		1990–2014	1989–2015	1987–2014	
Surgical Margin, N (%)	X		87 (22.7)	154 (18.8)	54 (14.5)	0.016
Adjuvant Radiotherapy, N (%)	X		24 (6.3)	34 (4.2)	10 (2.7)	0.050
African American, N (%)		X	103 (26.9)	160 (19.5)	59 (15.8)	.001

Summary statistics are provided prior to multiple imputation. Missing data is as follows: pre-surgery psa [17], pathological gleason score [3], SVI [18], lymph node [17], extracapsular extension [36], surgical margin [12], pgrade1 (476), pgrade2 (511). Complete data was available for surgery year and adjuvant radiotherapy

Using the Kattan score, 61 patients (19 obese, 29 overweight, and 13 normal weight) were placed into the high risk group, 333 patients (98 obese, 165 overweight, and 70 normal weight) into the medium risk group, and 1182 (266 obese, 626 overweight, and 290 normal weight) into the low risk group. The CaPSURE/CPDR score placed 75 patients (29 obese, 32 overweight, and 14 normal weight) into the high risk group, 473 (143 obese, 238 overweight, and 92 normal weight) into the medium risk group, and 1028 (211 obese, 550 overweight, and 267 normal weight) in the low risk group.

Hazard ratios (95% CI) indicated the relative risk of PCa recurrence for medium or high risk groups within each obesity group (Table 3). In both models, the interaction between risk group and obesity status was not statistically significant (Kattan *p* = 0.752, CaPSURE *p* = 0.147). In examining the Kattan score model within each obesity group, we found that, as expected, patients with medium and high risk scores had a higher risk of PCa recurrence compared to low risk patients. Similar results were observed in the CaPSURE/CPDR score model, with the exception that in the obese group, patients with high risk scores were not statistically different from those with low risk scores. Examining the high CaPSURE/CPDR column of Table 3, the difference in risk of recurrence in the high risk versus low risk groups is larger for normal weight patients than the same estimate in the obese patients. Hazard ratio estimates and confidence intervals from the observed data without imputation were similar to those presented in Table 3, with the exception that high risk scores were statistically different from those with low risk scores for obese men (data not shown).

**Table 3:** Hazard ratios (95% confidence interval) of PCa recurrence for medium and high risk groups compared to the low risk group by obesity status

Obesity Status	Medium Kattan	High Kattan	Kattan score^a^	Medium CaPSURE/CPDR	High CaPSURE/CPDR	CaPSURE/CPDR score^a^
Normal Weight	4.11 (2.20, 7.67)	13.2 (5.77, 30.1)	1.36 (1.25, 1.48)	3.83 (2.06, 7.14)	6.21 (2.60, 14.9)	3.16 (1.87, 5.34)
Overweight	2.89 (2.02, 4.13)	8.66 (4.71, 15.9)	1.37 (1.27, 1.46)	2.58 (1.77, 3.77)	4.64 (2.55, 8.44)	2.64 (1.89, 3.69)
Obese	2.56 (1.58, 4.15)	7.09 (3.54, 14.2)	1.32 (1.21, 1.44)	2.19 (1.26, 3.79)	1.89 (.920, 3.87)	1.15 (0.80, 1.65)

^a^Hazard ratio of PCa recurrence per 10-unit increase in risk score

**Fig. 1:**
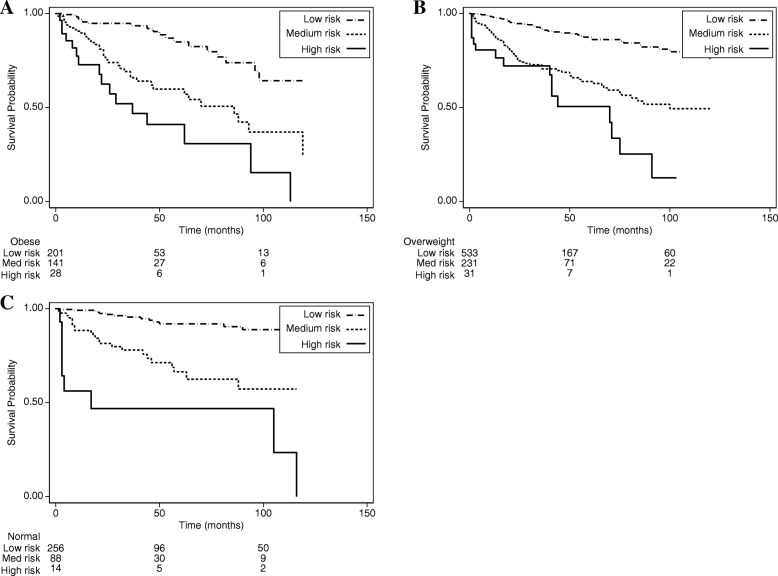
Estimated survival probability stratified by obesity status and risk groups (Kattan Score). **a** Obese patients. **b** Overweight patients. **c** Normal weight patients

**Fig. 2:**
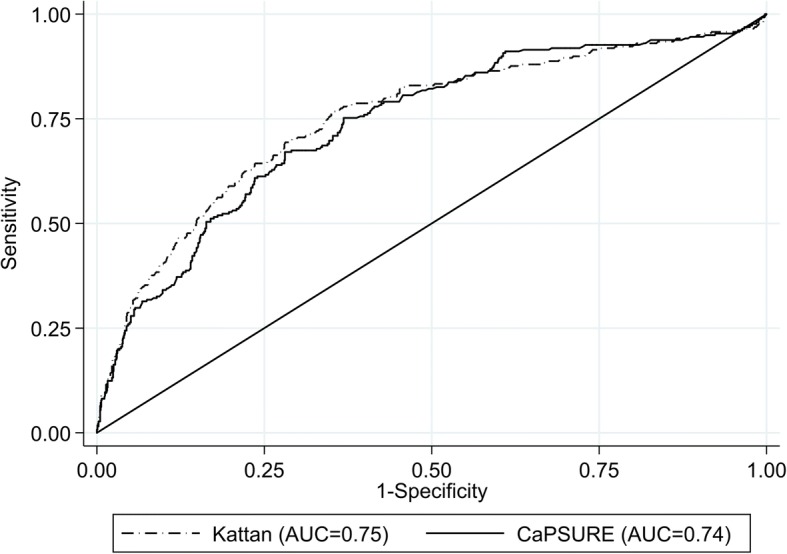
Receiver operating characteristics (ROC) curves for the accuracy of each risk score in predicting prostate cancer recurrence

Kaplan-Meier survival curves were used to compare the recurrence rates of patients by risk group and obesity group using the CaPSURE/CPDR model (Fig. 1). Consistent with results from Table 3, recurrence was more likely to occur in the medium and high risk groups compared to the low risk group (*p* < 0.001). However, within each PCa risk group, lower recurrence rates were observed for normal weight compared to obese men. Using AUC analysis, we also observed that the diagnostic accuracy for each risk score was comparable. The area under the curve is 0.75 (95% CI: 0.72, 0.79) for the Kattan score and 0.74 (0.70, 0.77) for the CaPSURE score. (Fig. 2) However, there was a suggestion of higher accuracy for the Kattan score in the normal weight group compared to the overweight and obese groups. These were not statistically significant differences. (Table 4 and Fig. 2).

**Table 4:** Area under the Curve (95% Confidence Interval) by Obesity Category for Each Risk Score

BMI Group	Kattan	CaPSURE
Normal weight	0.81 (0.72, 0.89)	0.76 (0.68, 0.84)
Overweight	0.73 (0.68, 0.78)	0.74 (0.69, 0.79)
Obese	0.75 (0.69, 0.81)	0.71 (0.64, 0.78)

## Discussion

This study is one of the first to examine how patient BMI may modify the accuracy of commonly used prediction tools for PCa recurrence. As expected, we observed an increase in the risk of PCa recurrence with an increase in the risk level of the prediction scores. We observed no statistically significant associations between risk score categories and the risk of PCa recurrence across BMI groups. However, we did observe a larger nonsignificant effect size among normal weight men (vs. obese) for PCa recurrence in high risk compared to low risk groups.

The obesity effect on PCa progression is controversial and has at times seemed conflicting. A recent large cohort study demonstrated that obesity was associated with a decreased risk of low grade PCa but an increased risk of high grade PCa [6]. Earlier analyses of the SCORE sample indicated that obesity (not stratified by PCa risk group) was significantly associated with advanced tumor stage and risk for biochemical recurrence among African American men, in particular [4]. A recent study of SCORE patients showed that obesity was associated with a significantly higher rate of pathologic upgrading of tumors, particularly among low and medium risk men [11]. Freedland et al. (2004) showed that moderately and severely obese men had a greater risk of biochemical failure than normal weight, overweight, or mildly obese men. After controlling for preoperative clinical factors and Gleason score, BMI remained a significant predictor. Moderately and severely obese men had a 4-fold increased risk of failure [23]. Efstathiou et al. (2007) showed that overweight and obese patients were almost twice as likely to die of prostate cancer as those with normal weight [24].

### PCa risk prediction

Widespread use of the PSA test has led to increased detection and treatment of clinically insignificant tumors. Historically, PCa treatments have been associated with risk of urinary and sexual dysfunction [25]. Therefore, high risk patients should be diagnosed as accurately as possible, since risk status influences patient treatment options and quality of life (both physical and psychological.) Even among low risk patients (eligible for active surveillance protocols), there is the potential for upgrading and upstaging to pathologically unfavorable disease [11, 25, 26].

Accurate estimates are essential for physicians to make recommendations for effective PCa management. Nomograms are the most appropriate estimates of prognosis because they maximize the incorporation of available, routinely collected, standardized prognostic information [27]. Although no nomogram predicts with 100% accuracy, they are the most accurate predictions of cancer outcomes that we have to date [13]. The accuracy of a nomogram may vary among patient populations, perhaps predicting outcomes better depending on risk group or patient characteristics [13]. Direct comparisons of predictive tools are needed to determine which nomograms are best suited for particular patient groups. The incorporation of novel biomarkers for PCa progression may increase predictive accuracy for some patients, particularly those at high risk for PCa recurrence [13, 14]. This increase in accuracy with biomarkers may not be observed among low risk patients [14]. However, the inclusion of BMI may have greatest utility among low and medium risk patients [11].

### Study limitations and strengths

One of our study limitations is that we use BMI to determine obesity. Other important measures of obesity, such as waist-to-hip ratio and percent lean body fat were unavailable for this secondary data analysis. However, BMI is one of the most clinically relevant measures of adiposity that we commonly use. Unlike other measures, BMI is readily available in the medical record and easily computed upon physical exam. BMI also predicts prostate cancer outcomes [4, 5, 28–30] and is associated with health outcomes in a trajectory similar to other measures of adiposity [31, 32].

Biochemical recurrence is not a perfect surrogate for poor PCa outcomes/fatality. However, biochemical recurrence is still clinically relevant as a decision point for adjuvant therapy among patients considered to be at increased risk for poor outcomes because of rising PSA after primary treatment.

Our study is limited by a small sample of high risk patients, so evidence is insufficient that our findings are generalizable to all radical prostatectomy patients. Interpretation of our results is also limited by the number of patients with missing data. There are differences reported between groups with missing data and those with complete data. This study is a cohort study based on secondary data analysis. Although more complete data collection would have been optimal, we were limited by the data available to us and overcame that limitation with state-of-the-science imputation methods. We used imputation methods to avoid the problem of having a very biased sample population which would be caused by including only patients with complete data. The original study also was not designed as a case-control study to investigate BMI associations. Rather, our patients were stratified by BMI at diagnosis, so our results reflect actual frequencies of overweight and obesity in a prostate cancer patient population. We believe that despite the limitations in study design, the manuscript still makes an important and novel contribution to the literature. Although we have a limited number of patients in our analyses, these results provide evidence that warrants building larger studies that examine the interplay between prognostic risk group, obesity, and PCa outcomes. We also have not been able to adjust our models for additional treatments after radical prostatectomy or other cause of death during the follow-up period. The availability of these data in the medical records was missing for many patients, perhaps because some patients were seen at other health systems for treatment during the follow-up period.

Additionally, our study likely was underpowered to detect interactions between obesity and risk groups. In a post-hoc power analysis using the observed risk groupings, we determined that, with good power, large effects comparing risk groups within an obesity category are detectable.

Other commonly used predictive tools, such as CAPRA-S score, were not studied in this project [33]. Future studies may include comparisons of nomograms to determine which has the best predictive ability for diverse PCa patients.

Given the high prevalence of obesity and PCa, the effect of obesity on PCa outcomes is a relevant clinical issue in men’s health. Our results suggest that the relationship between risk group and the time to PCa recurrence does not vary significantly by BMI group. This implies that patients with extremely severe cancer will expect poor outcomes, regardless of their obesity status. In cases of less severe cancer prognosis (lower PCa risk group), obese patients may be only slightly more likely to experience cancer recurrence. New studies are needed to determine if obesity modifies the utility of various prognostic tools. Clarifying the role of obesity in the interpretation and accuracy of PCa prediction tools will impact the selection of patients for PCa screening, treatment, and prevention of poor outcomes [34, 35].

## Conclusions

Determining which low risk patients are at risk for prostate cancer recurrence is important for clinical decision making, especially because the majority of PCa patients are diagnosed with localized disease and are likely to be in low risk prognostic groups [36]. Data are limited regarding the impact of predictive tools on medical decision making. Decision making may be facilitated when patients can see tailored predictions of their outcomes under various conditions [13]. Ultimately, PCa management can be improved for “low risk” obese patients who often present with unique disease features and may require a more aggressive treatment approach. Although we used imputation methods to enlarge the sample with complete data, our study results suggest that high risk status based on PCa nomogram scores may be most predictive among normal weight patients. Additional research in this area is needed.

## Abbreviations

AUC: area under the curve
BMI: body mass index
FCS: fully conditional specification
HR: Hazard Ratio
PCa: prostate cancer
SCORE: Study of Clinical Outcomes, Risk and Ethnicity
UPHS: University of Pennsylvania Health System
